# Vitamin D Deficiency in Women with Breast Cancer: A Correlation with Osteoporosis? A Machine Learning Approach with Multiple Factor Analysis

**DOI:** 10.3390/nu14081586

**Published:** 2022-04-11

**Authors:** Alessandro de Sire, Luca Gallelli, Nicola Marotta, Lorenzo Lippi, Nicola Fusco, Dario Calafiore, Erika Cione, Lucia Muraca, Antonio Maconi, Giovambattista De Sarro, Antonio Ammendolia, Marco Invernizzi

**Affiliations:** 1 Physical Medicine and Rehabilitation Unit, Department of Medical and Surgical Sciences, University of Catanzaro “Magna Graecia”, 88100 Catanzaro, Italy; nicola.marotta@unicz.it (N.M.); ammendolia@unicz.it (A.A.); 2 Operative Unit of Clinical Pharmacology, Mater Domini University Hospital, Department of Health Science, University of Catanzaro “Magna Graecia”, 88100 Catanzaro, Italy; gallelli@unicz.it (L.G.); desarro@unicz.it (G.D.S.); 3 Research Center FAS@UMG, Department of Health Science, University of Catanzaro “Magna Graecia”, 88100 Catanzaro, Italy; 4 Translational Medicine, Dipartimento Attività Integrate Ricerca e Innovazione (DAIRI), Azienda Ospedaliera SS. Antonio e Biagio e Cesare Arrigo, 15121 Alessandria, Italy; lorenzolippi.mt@gmail.com (L.L.); amaconi@ospedale.al.it (A.M.); marco.invernizzi@med.uniupo.it (M.I.); 5 Department of Health Sciences, University of Eastern Piedmont “A. Avogadro”, 28100 Novara, Italy; 6 Department of Oncology and Hemato-Oncology, University of Milan, 20126 Milan, Italy; nicola.fusco@unimi.it; 7 Division of Pathology, IEO, European Institute of Oncology IRCCS, 20141 Milan, Italy; 8 Physical Medicine and Rehabilitation Unit, Department of Neurosciences, ASST Carlo Poma, 46100 Mantova, Italy; dario.calafiore@asst-mantova.it; 9 Department of Pharmacy, Health and Nutritional Sciences, Department of Excellence 2018–2022, University of Calabria, 87036 Rende, Italy; erika.cione@unical.it; 10 Department of General Medicine, ASP 7, 88100 Catanzaro, Italy; lalumuraca@gmail.com

**Keywords:** vitamin D, breast cancer, osteoporosis, bone mineral density, machine learning, multiple factor analysis, cluster analysis

## Abstract

Breast cancer (BC) is the most frequent malignant tumor in women in Europe and North America, and the use of aromatase inhibitors (AIs) is recommended in women affected by estrogen receptor-positive BCs. AIs, by inhibiting the enzyme that converts androgens into estrogen, cause a decrement in bone mineral density (BMD), with a consequent increased risk of fragility fractures. This study aimed to evaluate the role of vitamin D3 deficiency in women with breast cancer and its correlation with osteoporosis and BMD modifications. This observational cross-sectional study collected the following data regarding bone health: osteoporosis and osteopenia diagnosis, lumbar spine (LS) and femoral neck bone mineral density (BMD), serum levels of 25-hydroxyvitamin D3 (25(OH)D3), calcium and parathyroid hormone. The study included 54 women with BC, mean age 67.3 ± 8.16 years. Given a significantly low correlation with the LS BMD value (r^2^ = 0.30, *p* = 0.025), we assessed the role of vitamin D3 via multiple factor analysis and found that BMD and vitamin D3 contributed to the arrangement of clusters, reported as vectors, providing similar trajectories of influence to the construction of the machine learning model. Thus, in a cohort of women with BC undergoing Ais, we identified a very low prevalence (5.6%) of patients with adequate bone health and a normal vitamin D3 status. According to our cluster model, we may conclude that the assessment and management of bone health and vitamin D3 status are crucial in BC survivors.

## 1. Introduction

Breast cancer (BC) is the most common malignancy in women and one of the leading causes of cancer-related death worldwide [1]. However, early detection of this tumor and the recent advances in cancer therapies have significantly improved patient overall survival, with a consequent rapid increase of BC survivors [2]. In this scenario, a critical issue described in the current literature is represented by the long-term consequences, with recent evidence focusing on physical and psychological sequelae affecting the quality of life of BC survivors [3,4].

In particular, osteoporosis is highly prevalent in post-menopausal BC survivors due its strict association with cancer treatments [5,6,7,8,9]. This specific condition is currently defined as cancer treatment-induced bone loss (CTIBL) and might be related to the hormone therapy that negatively affects bone mineral density (BMD) due to the reduction of residual serum endogenous estrogenic levels [5,8,10,11,12]. Moreover, chemotherapy has been related to an unspecific increase in bone resorption and a higher risk of fragility fractures [13,14,15]. Therefore, specific treatments preventing bone loss and reducing the risk of fragility fractures are strongly recommended to improve the long-term outcomes and management of BC patients [5,16].

A healthy lifestyle, including physical activity and a nutritional approach, is the cornerstone of a proper osteoporosis management [17,18]. On the other hand, it is well known that calcium and vitamin D3 supplementation could play a key role in maintaining bone health in BC patients [19,20]. Indeed, it has been reported that adequate levels of vitamin D3 might positively influence the risk of osteoporosis, physical performance and the risk of falls in older adults [9,20]. Moreover, several clinical trials, systematic reviews, and meta-analyses [21,22,23,24,25] reported significant advantages of the oral supplementation of calcium and vitamin D3 in reducing the fracture risk in elderly patients, with a reduction in the overall fracture risk ranging between 5% and 19%.

Vitamin D is a steroid compound with pleiotropic effects in the human body [26]. Though over 50 distinct vitamin D metabolites have been characterized so far, which has allowed us to articulate a whole vitamin D metabolome, only 1,25-dihydroxy vitamin D3 (1,25(OH)2D3) has been commonly identified as biologically active [27]. By agreement, the determination of the total level of 25(OH)D3 has been employed to estimate the vitamin D reserve. The physiological outcomes of further metabolites are only considered potential, as their roles in vivo remain disregarded [28]. Therefore, vitamin D status is an promising tool for predicting BC, dental and neurological diseases, and COVID-19 [29,30,31,32,33,34].

On the other hand, a few studies assessed the effects of calcium and vitamin D3 in preventing CTIBL in BC women [16]. According to the National Osteoporosis Foundation, the U.S. Preventative Services Task Force, the National Academy of Sciences, and the Institute of Medicine, women over 50 years old should receive 800–1000 IU of cholecalciferol per day [35], and the same dose is recommended for BC survivors at risk of CTIBL [36,37]. However, vitamin D3 deficiency remains largely prevalent in BC survivors due to both under-prescription and poor adherence to oral supplementation, with detrimental effects in terms of calcium homeostasis, skeletal metabolism and immune and cardiovascular systems’ functions [36]. 

Nearly half of the women diagnosed with BC are vitamin D-deficient [38], while prospective cohort studies have reported an inverse association between the serum levels of 25-hydroxyvitamin D3 (25(OH)D3) and breast cancer prognosis [39,40,41]. Indeed, low vitamin D levels have been found to be significantly associated with an increased risk of distant recurrence and early death in BC patients [42]. The pleiotropic effect of vitamin D, affecting the expression of at least 200 genes, is well known [43].

**Figure 1:**
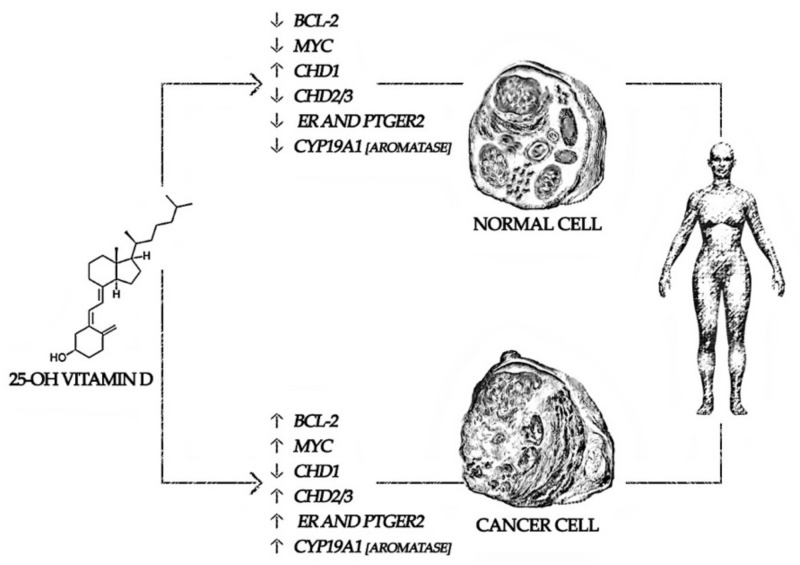
Differences in cholecalciferol pathways in breast cancer survivors. Gene abbreviation: BCL-2: B-cell lymphoma 2, CHD: Chromodomain-Helicase DNA-binding, CYP: Cytochrome P, ER: Estrogen receptor, MYC: MYeloCytomatosis, PTGER: Prostaglandin E Receptor.

Despite the mechanisms underpinning CITBL in BC survivors being far from full understanding, vitamin D3 could represent a molecular target in the complex pathological framework of BC osteoporosis [44,45]. The list of target genes that is common across cell models seems to be short, and the most clearly shared target is Cytochrome P (CYP24A1) [44]. High parathyroid hormone levels and hypercalcemia induce 1,25(OH)2D3 synthesis, stimulating the transcription of *CYP27B1* and increasing 1,25(OH)2D3 activity, with consequent down-stream action of *CYP27B1* and suppression of parathyroid hormone [46]. Moreover, the up-stream 1,25(OH)2D3-upregulated protein 1 attaches the disulfide-reducing protein thioredoxin and represses its capacity to inhibit reactive oxygen species. This unsuccessful inhibition of reactive oxygen species might in turn lead to stress-induced apoptosis [44,45,47], via B-cell lymphoma 2 (*BCL-2*), MYeloCytomatosis (*Myc*), and Chromodomain-Helicase DNA-binding (CHD) pathways, as depicted in Figure 1. 

The BCL-2 family consists of three subgroups: apoptotic promoters, apoptotic effectors, and anti-apoptotic proteins; indeed, their expression level and shifting status might determine a cell fate. These proteins, specifically BCL-2, BCL-XL, and MCL-1, have been associated with progression, chemoresistance, and metastatic potential in a range of cancers, including breast cancer [48,49]. Salehi-Tabar et al. demonstrated that 1,25(OH)2D3 could suppress the expression of c-Myc in vivo, and c-Myc protein levels were elevated in Vitamin D receptor (VDR)-deficient cells [50].

A growing interest in precision medicine approaches has been rising to treat several cancer conditions. More in detail, machine learning studies have successfully improved diagnostic capabilities in a wide range of medical applications [51,52]. To better understand the role of different variables in a statistical model, machine learning algorithms could need a more sophisticated approach [53]. Machine learning methods, such as k-Nearest Neighbors and Neural Networks, have been developed in recent years [54,55]. 

In this context, Multiple Factor Analysis (MFA) is considered a novel multivariant statistical approach allowing the analysis of several groups of continuous variables of different nature by clustering the study participants through a machine learning model [56,57]. Indeed, it weighs each variable with respect to the others and allows for clustering by diversifying individuals into different groups. 

These advances in machine learning might improve patient-tailored frameworks in both cancer diagnosis and treatment [58,59]. However, to date, few studies integrated emerging technologies for the patient-centered assessment of BC-related sequelae [60,61], and to the best of our knowledge, studies integrating machine learning approaches to evaluate the correlation of vitamin D3 and osteoporosis in BC women are lacking. 

Our hypothesis is that in in BC patients, in addition to a high frequency of vitamin D deficiency, there may be significant correlations of vitamin D deficiency with osteoporosis parameters. 

Therefore, in this study, we sought to assess the correlation between vitamin D deficiency and osteoporosis in BC women using a machine learning approach to deeply characterize the characteristics of BC survivors. 

## 2. Materials and Methods

### 2.1. Study Participants 

This observational cross-sectional study recruited women with BC referred to the Outpatient Service for Cancer Rehabilitation of the Physical Medicine and Rehabilitation Unit of the Azienda Ospedaliera “SS. Antonio e Biagio e Cesare Arrigo”, Alessandria, Italy. Patients were recruited over a 12-month period, from April 2021 to March 2022. The inclusion criteria were the following: (a) women in post-menopausal status, with a diagnosis of BC *ER*+; (b) hormone therapy; (c) surgery performed at least 12 months earlier. The exclusion criteria were the following: (a) T stage > 3; (b) age < 50 years; (c) evidence of major concurrent diseases; (d) patients undergoing treatment with corticosteroids, immunoglobulin or immunosuppressive drugs, and chemotherapy; (e) previous fragility fractures; (f) previous vitamin D3 supplementation. The study respected the Declaration of Helsinki and was approved by the local Ethical Committee (677/2021). All participants were asked to carefully read and sign an informed consent, taking precautions to protect the privacy of patients. Moreover, the study was performed in accordance with the “Strengthening the Reporting of Observational Studies in Epidemiology” (STROBE) Guidelines (https://www.equator-network.org/wp-content/uploads/2015/10/STROBE_checklist_v4_cross-sectional.pdf; accessed on 1 April 2021). 

### 2.2. Outcome Measures

The following demographic and anamnestic data were collected: sex, age, body mass index (BMI), smoking habit, BC situs (right/left), BC grading, type of breast surgery (conservative/mastectomy), adjuvant hormone therapy (tamoxifen or aromatase inhibitors). The following data regarding bone health were also assessed: lumbar spine (LS) BMD, LS Tscore, LS Zscore, femoral neck (FN) BMD, FN Tscore, FN Zscore, diagnosis of osteoporosis, diagnosis of osteopenia, serum levels of 25(OH)D3 (ng/mL), calcium (mg/dL), and parathyroid hormone (PTH) (pg/mL).

### 2.3. Multiple Factor Analysis

MFA is a multivariant statistical technique that allows the analysis of several groups of continuous variables of different nature, allowing the clustering of individuals via a machine learning model. It adopts a geometric approach based on a set of variables, vectorizing the inertia of each factor on the abscissa axis (dimension 1) and on the ordinate axis (dimension 2) [56]. The importance of the dimensions is given by the eigenvalue that indicates the highest percentage of variance on the Cartesian plot [62].

Once the nature of the dimensions with greater variance and inertia has been assessed, it is possible to evaluate how certain individual clusters are represented on a Cartesian model, formed by the aforementioned dimensions [63,64].

Based on this model, each study participant was positioned and classified into a definite group. Then, through the K-means clustering, we assigned the individuals to one of the groups (called clusters) based on the characteristics of the dataset, weighing the distance of each point using a Euclidean model applied to the machine learning approach [65]. Therefore, MFA might be considered as a factorization method in which bone health and anthropometric parameters influence the position of individuals, weighting their distance, characterizing certain clusters (osteoporosis, osteopenia, and normal bone health), and defining their vectors of influence a posteriori [57]. 

### 2.4. Data Management and Statistical Analysis

Statistical analysis was performed using R (v3.5.2 R Core Team, Vienna Austria). The continuous variables are presented as means ± standard deviations, and the categorical variables as medians and interquartile ranges. The Shapiro–Wilk test was performed to assess the distribution of all continuous data. Pearson’s correlation coefficients and regression analyses on parametric data assessed associations and correlations regarding the bone health status of the study participants and clinical and demographic features. A cut-off *p*-value of 0.05 was considered statistically significant. 

MFA was conducted in R–statistics software with “FactomineR” and “factoextra” package [66,67]. To validate MFA clustering, we performed K-means clustering as a machine learning algorithm, weighing each distance between two observations and evaluating the reliability of the definition of the different clusters [68]. By using the statistical software JASP v0.16 (JASP Team, Amsterdam, The Netherlands), we obtained the following scores: R^2^, a score that indicates the amount of variance explained by the model; the Akaike Information Criterion (AIC), where lower values represent better clustering outputs; the silhouette score, with value ranging from −1 to 1, where 1 represents dense clusters and well-separated data.

## 3. Results

Of the 58 subjects recruited, 4 did not match the inclusion/exclusion criteria and were excluded; thus, 54 BC women (mean age 67.3 ± 8.16 years) were included in the final analysis. The clinical characteristics of the patients enrolled are summarized in Table 1.

Regarding the bone health assessment, the mean LS BMD was 0.93 ± 0.17 g/cm^2^, whereas the mean FN BMD was 0.744 ± 0.10 g/cm^2^; 51.8% of the women with BC had a diagnosis of osteoporosis, and 42.6% had osteopenia. Furthermore, the mean serum level of 25(OH)D3 was 19.7 ± 7.2 ng/mL, and 52 patients (96.3%) reported hypovitaminosis D ([25(OH)D3] < 30 ng/mL). 

BC patients were stratified in four groups according to their 25(OH)D3 serum levels: 6 subjects with serum levels of [25(OH)D3] ≤ 9.9 ng/mL, 21 ones with serum levels of [25(OH)D3] between 10 and 19.99 ng/mL, 25 subjects with serum levels of [25(OH)D3] ≤ 9.9 ng/mL, and 2 patients with normal serum levels of [25(OH)D3] ≥ 30 ng/mL. Given these results, only 3.71% of the subjects demonstrated an optimal value of serum vitamin D3; curiously, patients with vitamin D3 below 30 ng/mL were normally distributed around the mean of 19.7 ng/mL. There were no significant differences among the four groups in all the variables considered, albeit a positive trend in terms of LS BMD (see Table 2). 

As reported in Table 3, no significant associations were found between mean serum levels of 25(OH)D3 and the continuous indices examined, except for a significant Pearson’s r of 0.30 obtained for the correlation with LS BMD. This slight correlation could be explained by the low BMD value in subjects with severe vitamin D3 deficiency (LS BMD = 0.740 ± 0.22 in patients with vitamin D3 levels ≤9.9 ng/mL).

### 3.1. Machine Learning Results

**Figure 2:**
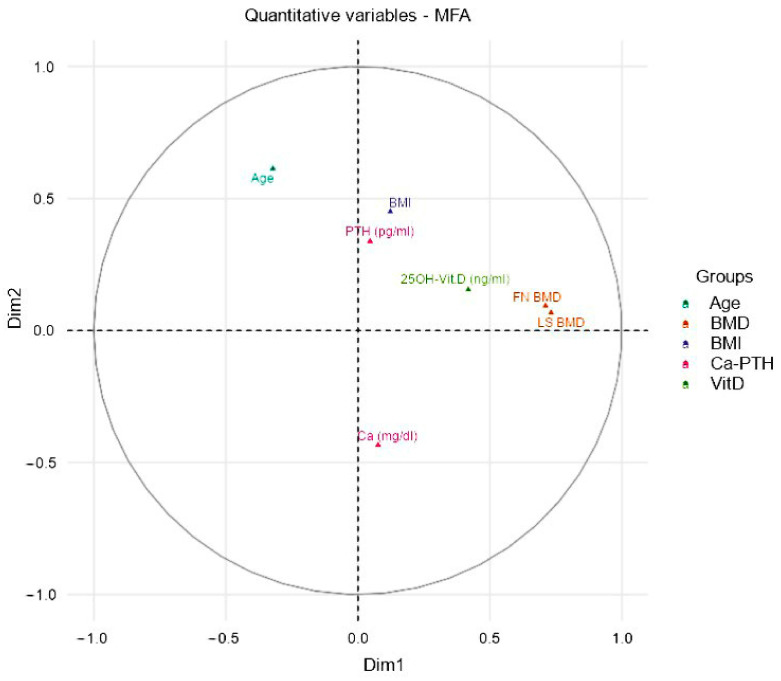
Correlations between quantitative variables and dimensions. The plot depicts the topographical influence in the arrangement of the variables on the graph along the abscissa (Dim1) and the ordinate (Dim2). Thus, we evaluated the weight of the single variables through crossed linear regressions, representing them two-dimensionally on a Cartesian plane. Therefore, the variables, indicated as vectors, according to the position in the represented circle, influence the spatial position of the individuals and consequently their clustering into groups. Abbreviation: BMD = Bone Mineral Density, FN = Femoral Neck, LS = Lumbar Spine.

Despite the low degree of association, to evaluate the nature of the dimensions and the influence of the variables, represented as vectors, we measured the eigenvalues and the variance of the model, contained in two dimensions (Cartesian axes). We reported an eigenvalue of 2.3 and a variance of 38.2% for Dimension 1 (abscissa) and an eigenvalue of 1.3 with 20.5% variance for Dimension 2 (ordinate); these data suggested that the first two dimensions explained 58.7% of the total inertia. Indeed, MFA evaluated the quantitative disposition of the single variables, thus showing a correlation of the analyzed variables on the two extracted dimensions. Positive factors are depicted on the plot together, whereas negative ones are arranged on opposite sides of the plot (see Figure 2 for further details.

We reported a positive arrangement for age, BMI, and PTH serum levels on dimension 2 (ordinates) and a positive arrangement for the FN BMD and LS BMD, as well as for vitamin D, on the axis of dimension 1 (abscissa). Therefore, greatest influence was attributed to the BMD values, whereas the possible positioning of an individual in the highest portion of the upper quadrant was correlated to an older age and higher BMI values 

**Figure 3:**
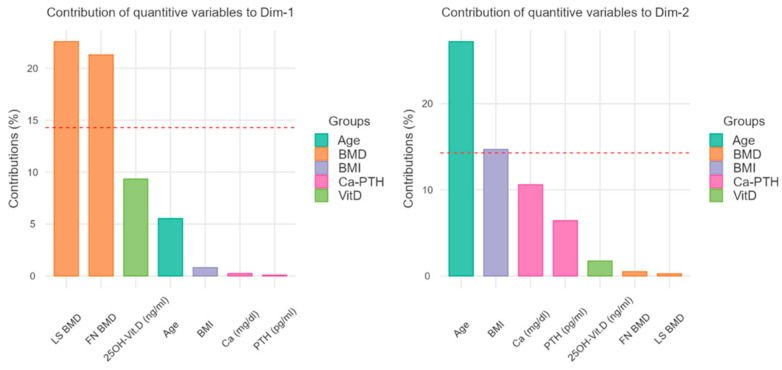
Contribution to the dimensions 1 and 2 (Cartesian axes). With the bar graphs, the plot contribution represents the weight of the single variables on the representativeness of the abscissa axis (Dimension 1) and of the ordinates (Dimension 2). As regards the horizontal axis of the previous figure, the greatest influence is attributed to the BMD values, while the vertical axis is related to the age and the BMI values. BMD = Bone Mineral Density, FN = Femoral Neck, LS = Lumbar Spine.

FN BMD and LS BMD could be considered as factors influencing the dimension 1, although not in a decisive way (LS: 22% and FN: 21%), as well as 25(OH)D3 serum levels. On the contrary, age and BMI could influence the position on the ordinates of the plot (see Figure 3 for further details).

**Figure 4:**
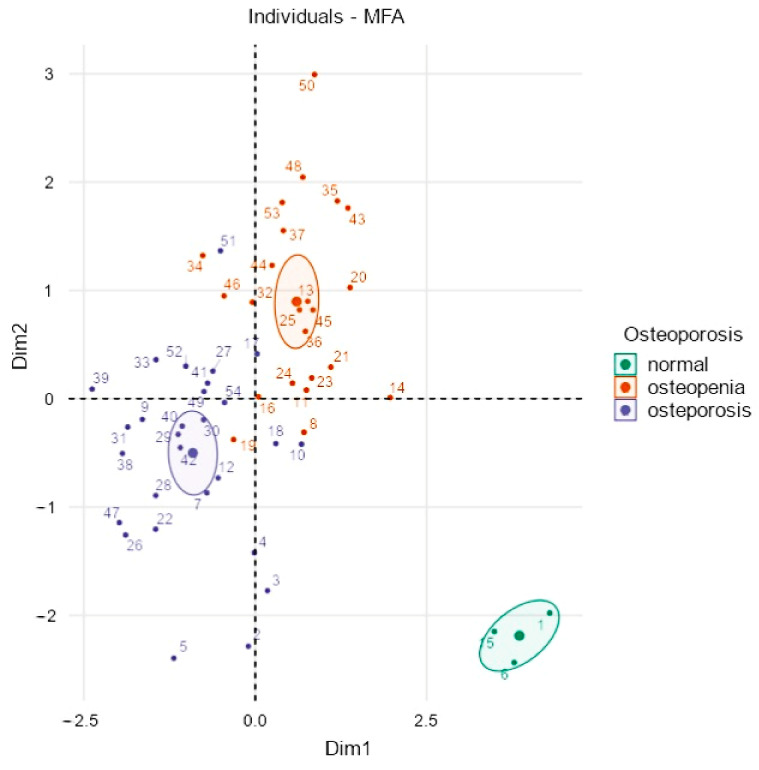
Clustered individual factor map. Each individual is positioned according to the Cartesian axes and thickens in specific clusters that reflect the influence of size along the horizontal axis for dimension 1 and along the ordinal axis for dimension 2. Normal subjects cluster at the bottom and right of the graph for the influence of age, BMI, and BMD, as previously described in Figure 2 and Figure 3.

Figure 4 depicts on a Cartesian model the disposition of the individuals and the consequent clusterization in three groups. More in detail, dimension 1 (abscissa) underlines the caliber of BMD and the serum levels of 25(OH)D3, whereas dimension 2 represents the negative correlation between age and a good bone health status. As shown in Figure 4, the non-osteopenic and non-osteoporotic group are positioned in the lower right quadrant of the graph, influenced by higher BMD and higher vitamin D3 serum levels from dimension 1 and lower age and BMI from dimension 2.

### 3.2. K-Means Clustering Model Analysis

We assessed the cluster quality by fitting the data to a K-means machine learning approach, obtaining k = 3 clusters for a dataset. Thus, the definition of three groups by MFA was confirmed through the analysis of the distance of each data point. Moreover, an R^2^ value of 0.54 was obtained, demonstrating that the model had a good reliability for a machine learning analysis. Moreover, an AIC of 976.45 showed a moderate quality of the model. Lastly, a Silhouette index of 0.34 demonstrated a low clustering trend for overlaps due to the small sample.

## 4. Discussion

This study aimed to evaluate the role of vitamin D3 deficiency in women with BC and its correlation with osteoporosis through a machine learning approach. At a first analysis of the reported data, there were no significant correlations between the mean serum level of 25(OH)D3 and bone health parameters and anthropometric data, except for a low significant positive correlation with the LS BMD value (r = 0.30; *p* = 0.025).

To date, osteoporosis is a disease characterized by low bone mineral density (BMD) and an increased risk for fragility fractures [69,70,71]. Our study reported a low percentage of patients with good bone health, low BMD indices, and a clear hypovitaminosis, outlining a prevalent CTIBL picture in these women. 

More in detail, 94.4% of our population showed a low BMD (51.8% had osteoporosis, and 42.6% had osteopenia). These results should be considered in the context of the small sample size investigated in the present work, although CTIBL is considered one of the most common long-term adverse events in BC survivors [16,72,73]. In this perspective, BMD decrease is mainly related to two determinants: hypogonadism onset due to chemotherapy and endocrine therapies and menopause-associated bone loss. 

Taken together, these two factors are responsible for osteopenia or osteoporosis occurrence and the increased fragility fractures risk in these women [16,72,74]. Thus, CTIBL should be early detected and properly treated, especially in BC survivors undergoing AIs, to prevent fragility fractures and improve the quality of life of these women [5,16,73]. The National Comprehensive Cancer Network (NCCN) guidelines recommend a daily oral intake of 1200 mg of calcium and 800–1000 IU of vitamin D3 for women at high risk for developing CTIBL [75]. Furthermore, several studies have recently expanded the evidence about the association of the *Apa1* polymorphism of *VDR* with post-menopausal osteoporosis and CTIBL [76,77,78,79]. This polymorphism could explain the previously observed significant correlation between 25(OH)D3 serum levels and LS BMD, as reported in this study.

Conventional statistical methods to assess the correlation between variables are quite reductive. In this context, Pearson correlation coefficient is only a linear correlation coefficient to measure the relationship between two variables [80,81,82], and for this reason, we decided to perform an MFA. This machine learning-based approach vectorizes all variables to arrange them on Cartesian axes built on the variable influences by clustering the individuals via their position along the trajectories [83]. 

The MFA model included about two-thirds of the variance; more in detail, the abscissa axis comprised 38.2% of the variance, while the ordinate axis comprised 20.5%, suggesting that, globally, the examined individuals were more influenced by Dimension 1, that is, the abscissa. This consideration could be described by the high contribution value of BMD regarding the abscissa axis (Dimension 1), but the 25(OH)D3 variable showed a similar vector that similarly contributed to forming clusters, as shown in Figure 2. Besides, the clustering of non-osteoporotic and non-osteopenic subjects could also be influenced by the contributions of Dimension 2 (ordinate axis), so differing in the lower right quadrant of Figure 4, compared to the other two clusters for the influence of lower age and lower BMI. In summary, the disposition of individuals appeared to be greatly influenced by Dimension 1 and, in particular, by BMD, but also by the serum levels of 25(OH)D3, as they were similarly arranged on the same clustering trajectory, as shown in Figure 2.

Decreased bone health and osteoporosis in women with BC are commonly due to hormonal therapy, and particularly post-menopausal women undergoing AIs are at high risk of developing osteoporosis [23,84,85]. Normal bone remodeling is under strict control, while in aging, menopause, and a cancer setting, there is a net loss of bone, sustained by the clinical mechanical stress of daily life activities on the molecular interactions among osteoclasts, osteoblasts, osteocytes, as well as by several systemic hormones regulating bone remodeling [19]. Estrogens are essential components in bone growth, intestinal absorption of calcium, bone resorption inhibition, and urinary calcium homeostasis and decrease by up to 90% in postmenopausal women [84,86]. Several studies have focused on identifying metabolites associated with BMD of different sites or with metabolic profiles of osteoporotic and low-BMD individuals categorized according to T-score or Z-score [70,87,88,89]. In this context, “metabolomics” could potentially provide the keys to understand the pathologic mechanism underpinning CITBL, providing new comprehensive CTIBL treatment approaches starting from prognostic markers such as metabolite changes [89,90,91]. For instance, bone loss mediated by estrogen deficiency is associated with the differentiation and activity of osteoclasts, which are in part related to the increased production of several cytokines including TNF-α, IL-1, and IL-6 that commonly led to a constant low-grade inflammation [92,93,94,95]_._ Estrogen receptors are extensively expressed in the gastrointestinal tract, and estrogens have been reported to increase *VDR* gene transcript level, protein expression, and endogenous 25(OH)D3 bioactivity in rat colonic mucosa. These factors may suggest that some of the estrogen activities in the colonic mucosa could be mediated, at least in part, by an increase in colonic mucosa responsiveness to endogenous 1,25-(OH)_2_D_3_ [96,97,98,99]. Remarkably, post-menopausal women with a vitamin D3 deficiency show higher concentrations of citrulline and ornithine than vitamin D3-deficient women with higher serum concentrations of BCAAs, glucogenic, and AAAs [22,87,92,100,101]. Lastly, both vitamin D3 and estrogen deficiency might have a negative impact on bone health in post-menopausal women with BC [77,99,102,103].

We are aware that our study has some limitations. First, our sample size was relatively small for an MFA, though it should be considered that we chose strict eligibility criteria. Second, any drop in measurement quality can prevent machine learning algorithms from accurately modeling the nonlinear association between features. However, it should be noted that we estimated almost two-thirds of the variance for the two dimensions. Third, several studies have focused on the identification of metabolites associated with low BMD at different sites or with the most disparate profiles of osteoporotic individuals, but to date we can only prospect association measures and prediction studies. Lastly, the assessment of serum vitamin D3 remains tied to an individual’s personal vitamin D3 response index rather than to the vitamin D3 status alone; there is a lack of data in terms of differences in the expression of the vitamin D3 receptor among the groups.

## 5. Conclusions

Taken together, our findings indicated a very low prevalence of patients with adequate bone health and a normal vitamin D3 status in a cohort of women with ER+ BC treated with AIs. A multiple factor analysis showed that both BMD and 25(OH)D3 serum levels influenced the arrangement design of the individuals on the same trajectories and, thus, in the construction of the clusters. Therefore, by this machine learning model, we may conclude that bone health and vitamin D3 status should be adequately assessed and treated to reduce the risk of fragility fractures in women with BC. 

## Figures and Tables

**Table 1:** Study population characteristics (*n* = 54).

Mean age (years)	67.29	±	8.16
BMI (kg/m^2^)	24.4	±	4.24
Smokers (*n*, %)	17		(31.48)
Grade 1 (*n*, %)	9		(16.66)
Grade 2 (*n*, %)	34		(62.96)
Grade 3 (*n*, %)	11		(20.37)
Surgery			
*Quadrantectomy* (*n*, %)	40		(72.07)
*Nodulectomy* (*n*, %)	1		(1.85)
*Lumpectomy* (*n*, %)	3		(5.55)
*Mastectomy* (*n*, %)	10		(18.51)
Radiotherapy (*n*, %)	43		(79.62)
Family history for osteoporotic fracture (*n*, %)	10		(18.51)
FN BMD (g/cm^2^)	0.744	±	0.10
FN T-score	−1.8	±	0.88
FN Z-score	−0.4	±	0.82
LS BMD (g/cm^2^)	0.930	±	0.17
LS T-score	−1.9	±	1.25
LS Z-score	−0.4	±	1.23
Osteopenia (*n*, %)	23		(42.59)
Osteoporosis (*n*, %)	28		(51.85)
[25OH-Vit.D] (ng/mL)	19.7	±	7.20
[25OH-Vit.D] <10 ng/mL (*n*, %)	6		(11.11)
[25OH-Vit.D] <20 ng/mL (*n*, %)	27		(50.00)
[25OH-Vit.D] <30 ng/mL (*n*, %)	52		(96.29)
Calcemia (mg/dL)	9.3	±	0.48
PTH (pg/mL)	44.7	±	12.94

Note: Continuous variables are expressed as means ± standard deviations, categorical variables are expressed as counts (percentages). Abbreviation: BMI = Body Mass Index, BMD = Bone Mineral Density, FN = Femoral Neck, LS = Lumbar Spine.

**Table 2:** Study population characteristics according to 25(OH)D3 serum levels.

	Overall (*n* = 54)			[25(OH)vit.D] ≤9.9 ng/mL (*n* = 6)			[25(OH)vit.D] = 10–19.99 ng/mL (*n* = 21)			[25(OH)vit.D] = 20–29 ng/mL (*n* = 25)			[25(OH)vit.D] ≥30 ng/mL (*n* = 2)		
Osteopenia (*n*, %)	23		(43)	1		(2)	12		(22)	8		(15)	2		(4)
Osteoporosis (*n*, %)	28		(52)	5		(9)	8		(15)	15		(28)	0		(0)
FN BMD (g/cm^2^)	0.744	±	0.10	0.737	±	0.12	0.720	±	0.12	0.762	±	0.08	0.798	±	0.01
FN T-score	−1.8	±	0.88	−2.3	±	0.54	−2.1	±	0.97	−1.7	±	0.87	−1.5	±	0.45
FN Z-score	−0.4	±	0.82	−1.2	±	1.04	−0.5	±	0.75	−0.2	±	0.80	−0.4	±	0.35
LS BMD (g/cm^2^)	0.930	±	0.17	0.740	±	0.22	0.938	±	0.17	0.967	±	0.15	0.965	±	0.30
LS T-score	−1.9	±	1.25	−2.4	±	0.79	−1.96	±	1.41	−1.7	±	1.23	−1.6	±	0.50
LS Z-score	−0.4	±	1.23	−1.7	±	0.83	−0.18	±	1.21	−0.1	±	1.19	−0.3	±	0.01
25OH-Vit.D T0 (ng/mL)	19.7	±	7.20	7.1	±	1.96	15.6	±	2.83	25.1	±	2.73	32.4	±	0.07
Calcemia (mg/dL)	9.3	±	0.48	9.2	±	0.41	9.2	±	0.52	9.3	±	0.52	8.9	±	0.35
PTH (pg/mL)	44.7	±	12.94	47.1	±	12.01	51.4	±	15.06	39.7	±	10.16	39.5	±	6.36

Continuous variables are expressed as means ± standard deviations, categorical variables are expressed as counts (percentages). Abbreviation: BMI = Body Mass Index, BMD = Bone Mineral Density, FN = Femoral Neck, LS = Lumbar Spine.

**Table 3:** Correlation between 25(OH)D3 serum levels and anthropometric characteristics and bone health parameters.

	r	*p* Value
LS BMD (g/cm^2^)	0.30	0.025 *
FN BMD (g/cm^2^)	0.14	0.300
Age (years)	−0.01	0.935
BMI (kg/m^2^)	0.06	0.654
Calcemia (mg/dL)	0.01	0.924
PTH (pg/mL)	−0.22	0.092

Note: Continuous variables are expressed as means ± standard deviations, categorical variables are expressed as counts (percentages). * *p* < 0.05. Abbreviation: BMI = Body Mass Index, BMD = Bone Mineral Density, FN = Femoral Neck, LS = Lumbar Spine.

## Data Availability

Dataset is available on request.
